# Effect of metabolic syndrome on coronary artery atherosclerotic plaque in type 2 diabetes mellitus patients

**DOI:** 10.3389/fendo.2025.1595475

**Published:** 2025-09-03

**Authors:** Yu-shan Zhang, Rui Shi, Yi-Ning Jiang, Yue Gao, Jin Wang, Yuan Li, Zhi-Gang Yang

**Affiliations:** Department of Radiology, West China Hospital, Sichuan University, Chengdu, Sichuan, China

**Keywords:** metabolic syndrome, type 2 diabetes mellitus, coronary computed tomography angiography, atherosclerotic, coronary artery plaque

## Abstract

**Background:**

The effect of MS on coronary artery plaques detected by coronary computed tomography angiography (CCTA) in type 2 diabetes mellitus (T2DM) patients is not fully understood. This study aimed to investigate the effect of MS and its components on coronary artery plaques by comparing CCTA characteristics, including plaque types, the severity of coronary plaques and high-risk plaques between T2DM patients with and without MS.

**Methods:**

This study retrospectively enrolled 2,431 patients with T2DM who underwent Coronary Computed Tomography Angiography (CCTA) at West China Hospital between January 2015 to February 2022. These patients were divided into two groups based on the presence or absence of metabolic syndrome (MS). The plaque type, coronary artery stenosis, extent of coronary artery plaques, high-risk coronary plaque features, the segment involvement score (SIS), the segment stenosis score (SSS) and multivessel disease (MVD) based on CCTA data were evaluated and compared between two groups.

**Results:**

For T2DM patients, those with MS (61.5%, n=1496) had more noncalcified/mixed plaques, more nonobstructive stenosis and higher SIS and SSS values than those without (P < 0.05 for all). The proportion of patients with any noncalcified plaque, any mixed plaque, SIS≥4 and SSS≥7were in parallel with the numbers of MS components (P for trend<0.01 for all). Multivariate logistic regression revealed that MS were independently associated with any noncalcified plaque (OR=1.232, P =0.024), any mixed plaque (OR=1.307, P=0.006), any nonobstructive stenosis(OR=1.615, P = 0.001), SIS≥4 (OR=1.529; P<0.001), SSS≥7 (OR=1.387; P=0.001), and any spotty calcification (OR=1.870, P =0.001) in T2DM patients after adjusting for the confounding factors.

**Conclusion:**

MS is independently associated with adverse coronary artery plaque characteristics in Type 2 Diabetes Mellitus (T2DM) patients, including increased mixed, noncalcified, nonobstructive, spotty calcification plaques, as well as extensive coronary artery disease (CAD). These findings highlight the need for early detection and management of MS to reduce cardiovascular risks in T2DM patients.

## Introduction

As a global health emergency of the 21st century, type 2 diabetes(T2DM) is growing at an alarming rate across all regions, imposing severe socioeconomic burden (1). Cardiovascular involvement significantly increases the risk of adverse events in diabetic patients (2). Previous trials of intensive glucose control have failed to reduce CVD in T2DM, suggesting a more complex pathophysiological than hyperglycemia alone (3). Therefore, exploration for other abnormal metabolic factors contributing to the progression of coronary atherosclerosis in T2DM patients is crucial.

Metabolic syndrome (MS) is characterized by a cluster of cardiovascular risk factors, including insulin resistance (IR) (42), impaired glucose tolerance, obesity, hypertension, and dyslipidemia, which collectively increase the risk of cardiovascular disease (4). MS significantly increases the risk of developing T2DM and major cardiovascular events by a factor of 5 and 3 respectively, and thus leading to an approximately 1.6-fold increase in mortality (5). Insulin resistance is known to play a central role in the pathogenesis of MS, and is also a primary cause of T2DM (6, 7), indicating a frequent coexistence between diabetes and metabolic syndrome. Previous studies using coronary computed tomography angiography (CCTA) to evaluate the adverse effects of MS on coronary artery plaques were limited to asymptomatic individuals or populations undergoing routine health examinations, and failed to evaluate high-risk plaque characteristics (8, 9). This study conducted a more comprehensive analysis of CCTA images in patients with type 2 diabetes mellitus (T2DM), exploring the impact of MS on plaque types, the severity of coronary plaques and high-risk plaques in T2DM patients, which could facilitate better risk stratification for diabetic patients with coexisting cardiovascular diseases.

## Methods

### Study population

This study is a single-center, retrospective, observational cohort study. A total of 3,372 hospitalized patients with type 2 diabetes (T2DM) at West China Hospital from January 2015 to February 2022 were included, all of whom underwent coronary computed tomography angiography (CCTA) within one week prior to admission or during hospitalization. Exclusion criteria (1): CCTA images with significant artifacts or poor quality (2); history of stent implantation, artificial valve replacement, or coronary artery fistula (3); missing critical clinical data, such as the data used for the diagnosis of MS; incomplete or missing clinical medical records. This retrospective study was approved by the ethics committee of our institution, which waived the requirement for informed consent. After applying these criteria, 2,431 patients were enrolled and categorized into MS group and non-MS group based on their adherence to the diagnostic criteria of MS.

T2DM was defined according to American Diabetes Association guidelines or treated with oral glucose-lowering agents or insulin (10). In accordance with modified National Cholesterol Education Program–Adult Treatment Panel III criteria (11), MS was diagnosed when at least three of the following conditions were present (1): waist circumference ≥ 90 cm in men and ≥ 80 cm in women, using the International Obesity Task Force criteria for the Asian-Pacific population to determine waist circumference criteria (2); triglyceride levels≥150 mg/dL (1.7 mmol/L) (3); HDL-cholesterol level < 40 mg/dL (1.0 mmol/L) in men and < 50 mg/dL (1.3 mmol/L) in women (4); blood pressure ≥130/85 mmHg or the use of antihypertensive medication; and (5) fasting glucose level ≥ 100 mg/dL (6.1 mmol/L) or the self-reported use of antidiabetic medication (insulin or oral agents) (11). For patients without waist circumference measurement, body mass index (BMI) was used instead of waist circumference and BMI > 25 kg/m^2^ was considered as exceeding the waist circumference threshold MS (12).

Hypertension was defined as having two consecutive systolic/diastolic blood pressure readings exceeding 140/90 mm Hg or the current use of antihypertensive medication. Dyslipidemia was diagnosed based on the presence of one or more of the following conditions: (1) hypercholesterolemia (TC ≥ 6.2 mmol/L), (2) hyper-LDL-C (LDL-C≥ 4.1 mmol/L), (3) hypertriglyceridemia (TG ≥ 2.3 mmol/L), and (4) hypo-HDL-C (HDL-C < 1.0 mmol/L in men and < 1.3 mmol/L in women) (13, 14). A history of smoking was recorded regardless of smoking cessation status, as was a history of alcohol consumption.

### CCTA scanning protocols

CCTAs indications, data acquisition and image post-processing were performed in accordance with the Society of Cardiovascular Computed Tomography guidelines (15). CCTA was performed using multiple-detector computed tomography (GE Healthcare, Waukesha, WI, USA) or multidetector CT systems (SOMATOM Definition, Siemens Medical Solutions, Forchheim, Germany; and SOMATOM Definition FLASH, Siemens Medical Solutions, Forchheim, Germany). Beta-blockers were not administered to lower the heart rate. The scan range extended from the tracheal bifurcation to 20 mm below the cardiac apex. All patients were positioned in the supine position and received an intravenous infusion of 70 to 90 ml (adjusted for body weight) of iodine contrast agent, followed by an injection of 30 ml of normal saline at the same flow rate. The Revolution CT system utilizes kV Assist and Smart-mA to automatically adjust tube voltage and tube current based on the patient’s scout image, with a collimation of 256 × 0.625 mm and rotation time of 0.28 s. The SOMATOM Definition system operates at a tube voltage of 100 ~ 120 kV, tube current of 220 mAs, collimation of 64/128 × 0.6mm, and rotation time of 0.33s. After the scan is completed, the initial dataset is immediately reconstructed, and the highest-quality images are transferred to a post-processing workstation (Syngo-Imaging, Siemens Medical Solution Systems, Forchheim, Germany) for image analysis. When plaques are highly calcified, Sinogram Affirmed Iterative Reconstruction (SAFIRE) is utilized to reduce image noise and optimize image quality. Coronary artery plaques are evaluated using maximum intensity projection, multiplanar reconstruction, curved planar reconstruction, and volumetric reconstruction.

### Image analysis

**Figure 1:**
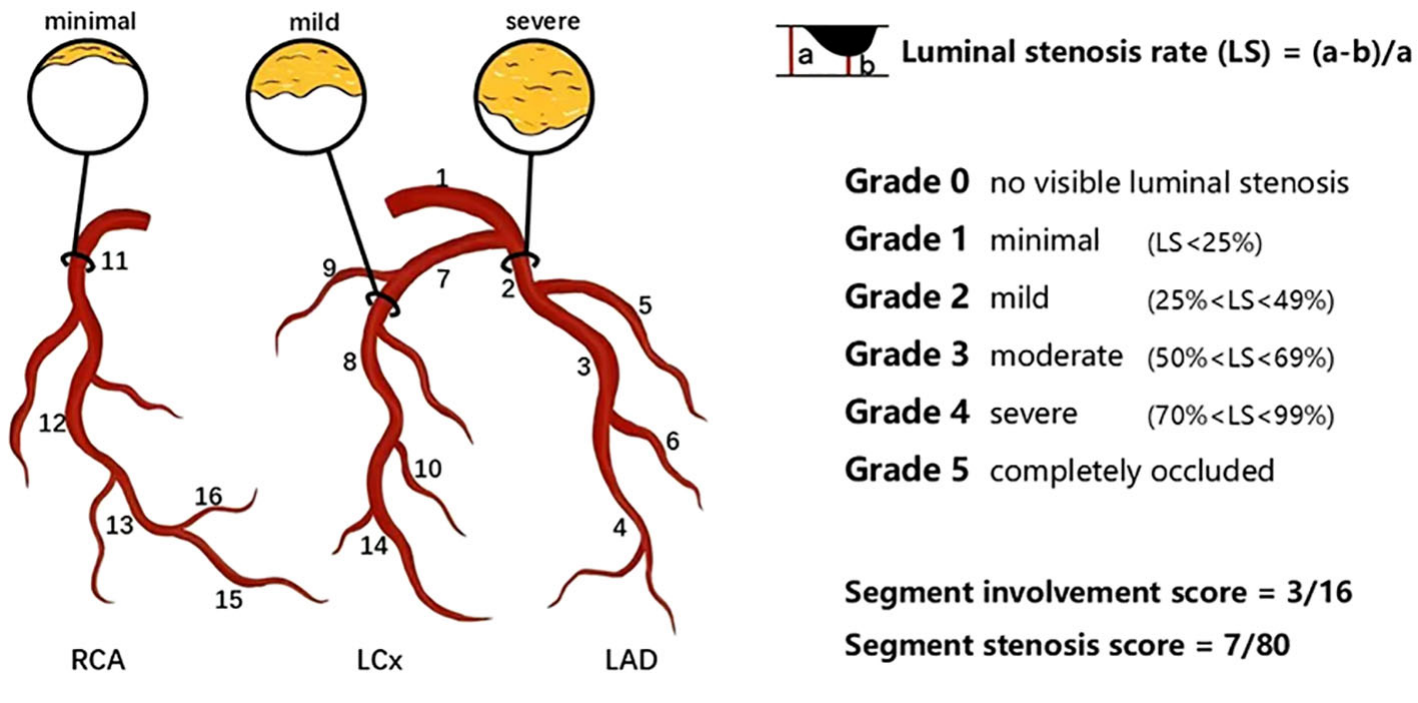
Schematic diagram of the degree of coronary artery stenosis and coronary artery segmentation. In this example, plaques distribute on proximal RCA, mid-LAD and proximal LCx. SIS was calculated by the number of coronary artery segments observed with plaques, which was 3 out of a possible 16 in this example. SSS was calculated by the minimal plaque in the proximal RCA (scored 1), mild plaque in the mid-LAD (scored 2) and severe plaque in the proximal LCx (scored 4). Thus, the SSS was 7 out of a possible 80. LAD left anterior descending artery; LCx left circumflex; RCA right coronary artery; SIS segment involvement score; SSS segement stenosis score. 1 left main coronary artery; 2 proximal LAD; 3 mid-LAD; 4 distal LAD; 5 first diagonal branch; 6 second diagonal branch; 7 proximal LCx; 8 distal LCx; 9 first obtuse marginal branch; 10 second obtuse marginal branch; 11 proximal RCA; 12 mid-RCA; 13 distal RCA; 14 left posterolateral artery; 15 right posterolateral artery; 16 posterior descending artery.

The plaque type, coronary artery stenosis, extent of coronary artery plaques and high-risk coronary plaque features based on CCTA data was qualitatively analyzed by two professional cardiologists who were masked to the clinical results and group identities. For segment-wise analysis, coronary artery trees were divided into 16 separate segments according to the revised standards of the American Heart Association (**Figure 1**) (16, 17). Each plaque was categorized based on its composition as (a) calcified plaque (plaques with higher density than contrast-enhanced lumen); (b) non-calcified plaque (plaques with lower CT attenuation than contrast-enhanced lumen, no calcification);(c) Mixed plaques (calcified with non-calcified components in a single plaque) (18). The severity of lumen stenosis caused by detected plaques was quantified and graded as a 5-point scale based on the Coronary Artery Disease(CAD)-Reporting and Data System: Grade 0, no visible luminal stenosis; Grade 1, lumen stenosis < 25%; Grade 2, lumen stenosis 25-49%; Grade 3, lumen stenosis 50-69%; Grade 4, lumen stenosis 70-99%; Grade 5, completely occluded (19). Obstructive stenosis was defined as lumen stenosis ≥50%. SIS represented the sum of coronary artery segments involved by plaque, with each segment’s plaque and lumen stenosis recorded as 1 point (0–16 points), which indicates the extent of coronary plaque involvement. SSS was defined as the sum of the stenosis scores of the relevant stenosis grades of all segments for each patient (0–80 points), which indicates the degree of stenosis of the coronary arter of coronary artery (16, 20). According to the American College of Cardiology/American Heart Association guidelines, multivessel obstructive disease (MVD) was defined as the presence of more than one vessel with stenosis ≥ 70% or LM stenosis ≥ 50% (21). The high-risk plaque features comprised of low-attenuation noncalcified plaque, positive remodeling, spotty calcification and “napkin ring” signs. The low-attenuation noncalcified plaques was defined as areas within plaques >1 mm^2^ with CT values <30 Hounsfield Units (HU). The remodeling index (RI) was defined as the ratio of the maximum vascular diameter at the lesion site (including plaque and lumen) to the diameter of the normal proximal lumen (arterial remodeling index = lesion plaque area/reference area). A RI of ≥ 1.1 indicates positive remodeling (outward expansion of the vessel wall). Spotty calcification was characterized as small focal calcifications of <3mm in any direction with a length diameter of < 3 mm in any plane within a non-calcified plaque, with a length diameter less than 1.5 times the vessel diameter and a short diameter of less than 2/3 the vessel diameter. The napkin-ring sign was described as a plaque core with low CT attenuation surrounded by an annular area with slightly higher CT attenuation (22–24).

### Statistical analysis

The baseline clinical and imaging data of the patients were stratified based on the presence of MS, and comparisons were made between the MS group and non-MS group in terms of differences in clinical baseline features and multi-row CT findings. Categorical variables were presented as number (%) and compared using a Chi-Square test. Continuous variables with normal distribution, such as age, height, weight, etc., were expressed as mean ± standard deviation and analyzed using student’s t-test. Non-normally distributed continuous variables, such as number of patches, were presented as median (interquartile range) and analyzed using Wilcoxon rank sum tests. **χ^2^** tests with linear-by-linear associations were applied to examine the significance of any linear trend of presence of any coronary artery plaque and extensive coronary plaques according to the number of MS components. Multivariate logistic regression was employed to analyze the relationship between coronary artery disease and MS along with other common cardiovascular risk factors. A two-tailed P value less than 0.05 was considered statistically significant.

## Results

### Study population

A total of 3349 T2DM patients were included in this study in the beginning, and after using exclusion criteria 2431 participants (935 patients without MS, 1496 patients with MS) were studied. The main clinical characteristics of the subjects were compared according to the presence or absence of MS (**Table 1**). The mean age of the participants was 69.2 ± 10.6 years, and 63% were male. The patients in the MS group had higher weight (non-MS vs. MS: 60.1 ± 9.6 vs. 69.1 ± 12.6, P<0.001) and body mass index (BMI, non-MS vs. MS: 22.6 ± 2.60 vs. 26.4 ± 10.7, P< 0.001) values. The MS group showed a higher prevalence of hypertension (46% vs. 83%, P<0.001), dyslipidaemia (53% vs. 88%, P< 0.001) and more frequent use of statin (38% vs. 44%, P= 0.004) and Biguanides (23% vs. 30%, P< 0.001). The level of cystatin C (CysC), high-density lipoprotein cholesterol (HDL-C) and plasma triglyceride (TG) was higher in patients with MS than those without MS (p<0.05 for all). There were no significant differences observed in sex, height, smoking history, drinking history, the use of insulin, the use of α-Glucosidase inhibitor and other laboratory measures between the two groups (p>0.05).

**Table 1:** Baseline characteristics of study population.

	Non-MS (n= 935)	MS (n =1496)	p
Variables Clinical parameters			
Age, years	69.12 ± 10.23	67.62 ± 10.84	0.001
Male (%)	604 (65%)	927 (62%)	0.198
Height, cm	162.84 ± 8.19	162.68 ± 9.52	0.686
Weight, kg	60.13 ± 9.57	69.12 ± 12.60	**<0.001**
BMI, kg/m^2^	22.59 ± 2.60	26.35 ± 10.72	**<0.001**
SBP, mmHg	133.76 ± 19.06	138.96 ± 20.33	**<0.001**
DBP, mmHg	77.54 ± 11.44	80.19 ± 12.44	**<0.001**
Hypertension (%)	429 (46%)	1240 (83%)	**<0.001**
Dyslipidemia (%)	497 (53%)	1314 (88%)	**<0.001**
Smoking History	935 (40%)	557 (37%)	0.227
Drinking History	935 (27%)	439 (29%)	0.224
Use of Statin, n (%)	358 (38%)	661 (44%)	**0.004**
Use of Insulin, n (%)	234 (25%)	404 (27%)	0.226
Use of Biguanides, n (%)	218 (23%)	445 (30%)	**<0.001**
Use of α-Glucosidase inhibitor, n (%)	181 (19%)	235 (16%)	0.077
Biochemical parameters			
Glucose, mg/dl	7.51 ± 3.13	7.75 ± 2.95	0.058
UREA, mg/dl	6.16 ± 2.65	6.53 ± 5.31	0.050
Cr, mg/dl	92.07 ± 220.45	117.35 ± 459.09	0.121
TC, mmol/L	4.09 ± 1.03	4.09 ± 1.21	0.996
TG, mmol/L	1.10 ± 0.44	2.00 ± 1.56	**<0.001**
HDL, mg/dl	1.34 ± 0.33	1.01 ± 0.29	**<0.001**
LDL, mg/dl	2.34 ± 0.90	2.34 ± 0.95	0.935
CysC, mg/dl	1.10 ± 0.60	1.25 ± 1.20	**<0.001**
CK, IU/L	117.02 ± 205.77	107.66 ± 181.61	0.248
LDH, IU/L	180.10 ± 59.30	178.15 ± 57.63	0.429

Data are presented as mean±SD or number (percentage). P values in bold are < 0.05.

BMI, body mass index; SBP, systolic blood pressure; DBP, diastolic blood pressure; TG, triglycerides; TC, total cholesterol; HDL, high-density lipoprotein; LDL, low-density lipoprotein; CysC, Cystatin C; CK, Creatine kinase; LDH, Lactate dehydrogenase.

### CCTA findings in non-MS and MS group

**Figure 2:**
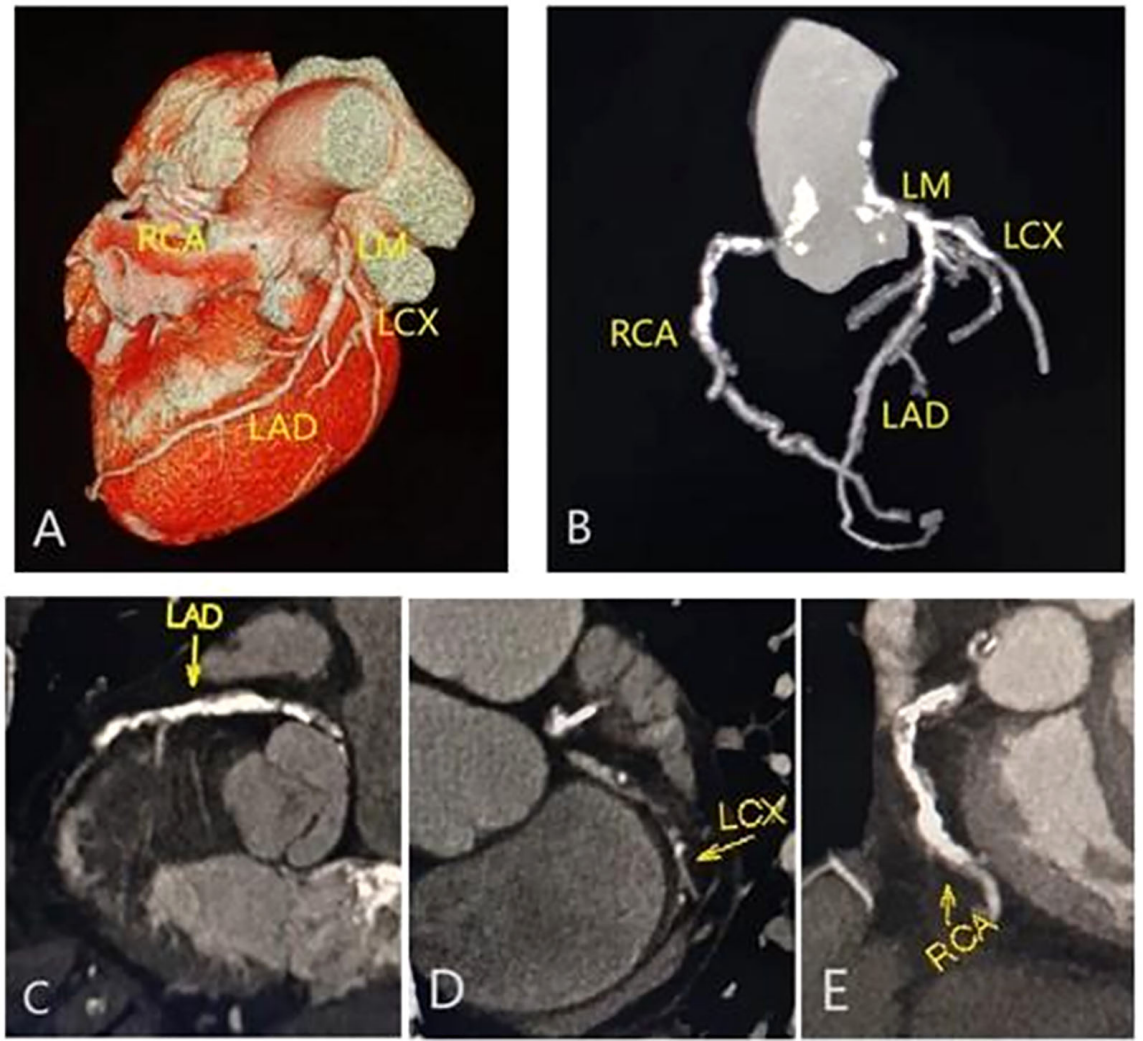
|Representative CCTA images of multivessel disease in a male with type 2 diabetes mellitus and metabolic syndrome. Volume rendering image **(A)**; maximum intensity projection (MIP) CT image **(B)**; and curvature plane reconstruction images **(C-E)** show the non-smooth edges, diffuse calcified, and mixed plaques of the LM, LAD, RCA and LCX.

**Figure 3:**
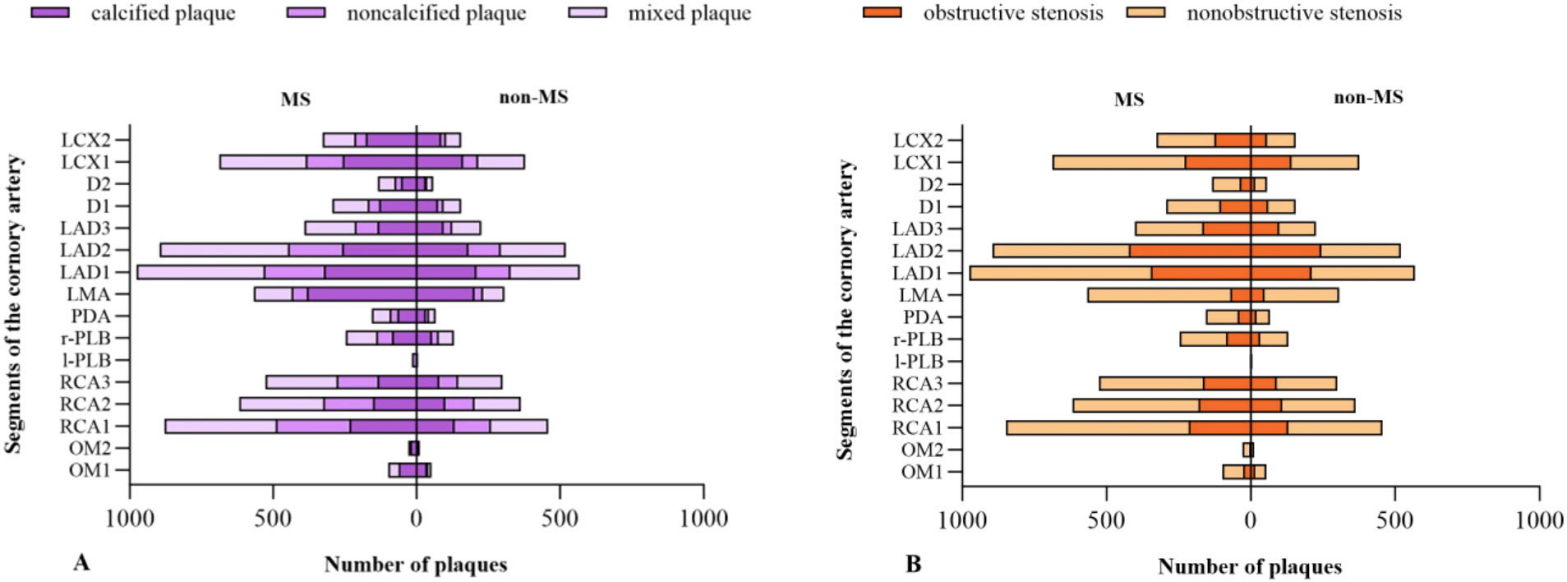
Anatomical distribution of plaques in different groups. Distribution of different types of plaque **(A)**; Obstructive and nonobstructive stenosis **(B)**.

A total of 10549 coronary plaques were analyzed [non-MS vs. MS 3752 vs. 6797]. The plaque types, coronary artery stenosis, extent of coronary artery plaques, and high-risk coronary plaque features between MS and non-MS groups were compared in **Table 2**. Regarding plaque types, the MS group exhibited a higher number of mixed plaques and noncalcified plaques compared to the non-MS group [non-MS vs. MS mixed plaques: 1.7 ± 2.3 vs. 2.0 ± 2.6, P =0.001; noncalcified plaques: 0.8± 1.2 vs. 0.9 ± 1.3, P values=0.004] (**Figure 2**, **Table 2**). No significant difference observed in the number of calcified plaques between two groups(p>0.05). The MS group had a higher proportion of patients with any noncalcified plaques than the non-MS group [non-MS vs. MS: 43.2% vs. 49.3%, P < 0.001] (**Table 2**). There were no significant differences in the proportion of patients with any calcified plaque or mixed plaque in between the MS and non-MS groups, nor in the proportion of patients with obstructive CAD (P > 0.05). The distribution of plaque types and high-risk plaque features within the coronary tree for the different groups is illustrated in **Figure 3**.

**Table 2:** Characteristics of coronary artery plaques detected by CCTA in DM patients with and without MS.

	Non-MS- (n = 935)	MS (n =1496)	P value
Plaque type			
Calcified plaque	1.58 ± 2.02	1.66 ± 2.05	0.352
Mixed plaque	1.66 ± 2.30	1.95 ± 2.58	**0.001**
Noncalcified plaque	0.76 ± 1.15	0.93 ± 1.26	**0.004**
Stenosis caused by plaques			
Obstructive Stenosis	1.37 ± 2.23	1.50 ± 2.78	0.178
Nonobstructive Stenosis	2.64 ± 2.19	3.04 ± 2.30	**<0.001**
Extent of the coronary plaque			
SIS	4.01 ± 3.32	4.54 ± 3.43	**<0.001**
SSS	8.65 ± 8.99	9.67 ± 9.30	**0.008**
High-risk plaque feature			
Low-Attenuation Noncalcified Plaque	0.29 ± 0.82	0.31 ± 0.79	0.629
Positive remodeling	0.32 ± 0.85	0.35 ± 0.78	0.352
Spotty Calcification	0.19 ± 0.62	0.25 ± 0.73	0.028
Napkin-Ring sign	0.03 ± 0.22	0.04 ± 0.25	0.564
Any Calcified plaque	538 (57.5%)	919 (61.4%)	0.057
Any Mixed plaque	501 (53.6%)	857 (57.3%)	0.074
Any Noncalcified plaque	404 (43.2%)	738 (49.3%)	**0.003**
Multivessel disease	79 (8.4%)	129 (8.6%)	0.882

Data are presented as the mean ± SD or number (percentage).

SIS, segment involvement score; SSS, segment involvement score.

Bold text represents a category, and the non-bold text below it indicates items that belong to this category. For example, plaque types are divided into three categories: calcified plaque, non-calcified plaque, and mixed plaque.

P values in bold are < 0.05.

Regarding the coronary artery stenosis, nonobstructive stenosis was more frequently observed in patients with MS than those without [non-MS vs. MS: 2.6 ± 2.1 vs. 3.0 ± 2.3 P < 0.001]. No significant difference showed in the number of obstructive stenosis between the MS and non-MS group (p>0.05). Regarding the high-risk plaque features, the subjects in the MS group had a higher prevalence of spotty calcification compared to the non-MS group [non-MS vs. MS: 0.2 ± 0.6 vs. 0.3 ± 0.7, P=0.028] (**Table 2**).

The subjects in MS group had higher SIS and SSS scores compared to those in the non-MS group [non-MS vs. MS SIS: 4.0 ± 3.3 vs. 4.5 ± 3.4 P values< 0.001; SSS: 8.7 ± 9.0 vs. 9.7 ± 9.3, P =0.008]. The difference in the proportion of subjects with multivessel disease between the MS and non-MS group was not statistically significant (p>0.05).

### The association between the number of MS components and coronary artery atherosclerosis

**Figure 4:**
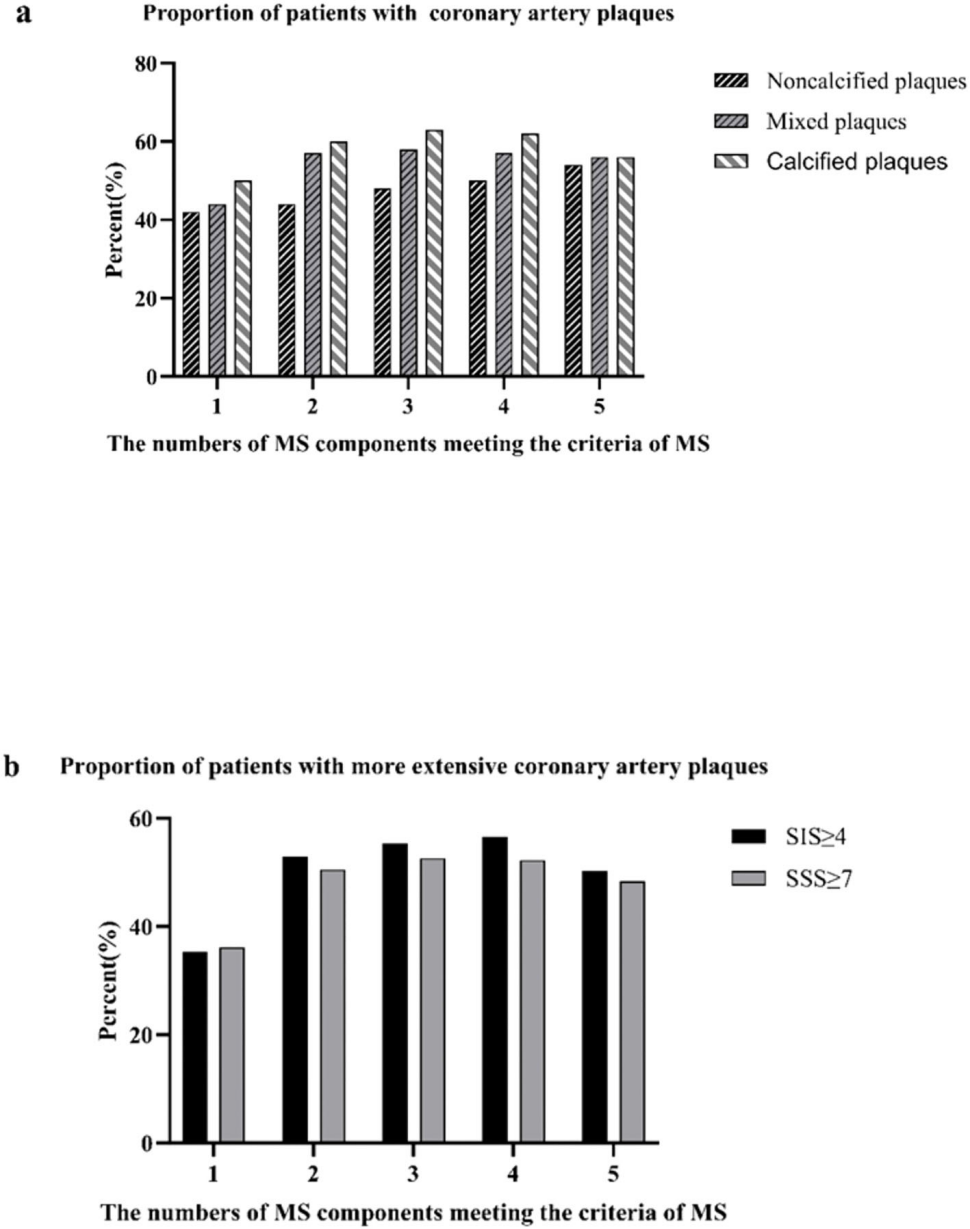
|Proportions of patients with coronary artery atherosclerosis according to the number of the metabolic syndrome (MS) components present **(a)**; proportion of patients with coronary artery plaques proportions of patients with extensive coronary artery plaques **(b)**.

The correlation between the number of MS components and coronary artery atherosclerosis was showed in **Figure 4**. As the number of MS components increased, so did the proportion of patients with noncalcified/mixed plaques, SIS≥4 and SSS≥7. The percent of patients with noncalcified plaques increased gradually from 42% in subjects without any MS component to 54% in those with three components, and then maintained relatively stable (P for trend <0.001; **Figure 4**). A similar trend was observed in the association between MS components and the proportion of patients with mixed plaques, SIS≥4 and SSS≥7 (mixed plaques: P for trend <0.001; SIS≥4: P for trend <0.01; SSS≥7: P for trend <0.01. **Figure 4**).

### Multivariate logistic regression analysis for variables associated with CCTA findings

Multivariate logistic regression analysis was performed to determine if MS was an independent risk factor for the presence of coronary artery plaques, extensive coronary artery plaques and high-risk plaque features. After adjustment for confounding factors including age, sex, SBP, etc., MS was found to be an independent risk factor for the presence of any mixed plaque, any noncalcified plaque and any nonobstructive stenosis [non-MS vs. MS, any mixed plaque: odds ratio=1.232, P=0.024; any noncalcified plaques: odds ratio=1.307, P=0.006; and any nonobstructive stenosis: odds ratio=1.615, P=0.001] (**Table 3**). MS was also significantly associated with more extensive and severe CAD (SIS≥4 and SSS≥7) and spotty calcification plaques after adjusting for the same confounding factors [non-MS vs. MS, SIS≥4: odds ratio=1.529, P=0.024; SSS≥7: odds ratio=1.387, P=0.006; and spotty calcification: odds ratio=1.870, P =0.001] (**Table 3**).

**Table 3:** Multivariate logistic regression analysis for variables associated with CCTA findings.

	Any noncalcified plaque		Any mixed plaque		Any nonobstructive stenosis	
	Odds ratio	P value	Odds ratio	P value	Odds ratio	P value
Age	1.028	.105	1.038	**.001**	1.064	**.001**
Sex	1.229	.052	1.612	**.001**	1.769	**.001**
SBP	1.004	.060	1.006	.003	1.008	**.008**
Smoking	1.211	.091	1.231	.077	1.440	**.027**
MS	1.232	**.024**	1.307	**.006**	1.615	**.001**

	SIS≥4		SSS≥7		Any spotty calcification	
	Odds ratio	P value	Odds ratio	P value	Odds ratio	P value
Age	1.046	**.001**	1.044	**.001**	1.023	**.001**
Sex	1.556	**.001**	1.584	**.001**	1.644	**.002**
SBP	1.006	**.007**	1.005	**.016**	1.005	**.021**
Smoking	1.270	**.043**	1.317	**.019**	1.412	**.027**
MS	1.529	**.001**	1.387	**.001**	1.870	**.001**

Adjusted model: Adjusted for age, sex, SBP, drinking history, smoking history, BMI, TC and LDL.

BMI, body mass index; SBP, systolic blood pressure; DBP, diastolic blood pressure; TC, total cholesterol; LDL, low-density lipoprotein.

Data in parentheses are 95% confidence intervals. Factors with a P value of less than 0.1 remained in the models. P values in bold are < 0.05.

We performed the same analysis to compare the effects of different components of MS on coronary atherosclerosis, the results of which are shown in **Table 4**. After adjusting for confounding factors, abnormal level of HDL and TG did not have statistically significant effects on most CCTA results(p>0.05); only HDL had significant effects on the presence of spotty calcification plaques [non-MS vs. MS, odds ratio=1.411, P=0.010]. Obesity is an independent factor for the presence of obstructive stenosis, low-attenuation plaques, SSS≥7, and multiple vessel disease. Hypertension is an independent factor for calcified/noncalcified plaques, obstructive/nonobstructive stenosis, SIS≥4, SSS≥7, most high-risk plaques, and multivessel disease, and it also exhibited a higher odds ratio than obesity.

**Table 4:** Multivariate logistic regression analysis for different components of MS associated with CCTA findings.

	HDL		TG		Obesity		Hypertension	
	Odds ratio	P value	Odds ratio	P value	Odds ratio	P value	Odds ratio	P value
Any Calcified Plaque	1.053	.551	.879	.169	1.138	.157	1.577	**<.001**
Any Mixed Plaque	1.182	.359	.913	.328	1.118	.213	1.172	.063
Any Noncalcified Plaque	1.175	.083	1.041	.659	.850	.062	1.334	**.016**
Any Obstructive stenosis	1.050	.573	1.001	.993	1.253	**.011**	1.617	**<.001**
Any Nonobstructive stenosis	1.287	.050	.901	.405	.886	.321	1.850	**<.001**
Any Low-Attenuation Noncalcified Plaques	1.083	.479	1.165	.199	1.292	**.027**	1.272	**.003**
Any Positive Remodeling	1.027	.796	1.270	.054	1.210	.075	1.533	**.007**
Any Spotty Calcification	1.411	**.010**	1.049	.714	1.197	.151	1.718	**.007**
Any Napkin-Ring sign	.757	.308	1.356	.285	1.708	.067	2.062	.131
Segment involvement score ≥ 4	1.107	.237	.942	.520	1.131	.171	2.123	**<.001**
Segment stenosis score ≥ 7	1.108	.232	.937	.482	1.214	**.030**	2.052	**<.001**
Any multiple vessel disease	.995	.999	1.071	.523	1.354	**.002**	1.642	**.004**

Adjusted model: Adjusted for age, sex, SBP, drinking history, smoking history, TC and LDL.

P values in bold are < 0.05.

## Discussion

This retrospective study demonstrated that compared to patients without MS (non-MS group), those with T2DM and concurrent MS displayed a significantly higher burden of coronary plaques. Specifically, these patients exhibited a higher prevalence of noncalcified/mixed plaques, a greater number of high-risk plaques, more extensive and severe CAD. Furthermore, the proportion of patients with noncalcified/mixed plaques and extensive coronary plaques showed a positive association with the numbers of MS components. Multivariate analysis showed that MS was independently associated with more extensive and severe CAD. This association extended to the occurrence of mixed, noncalcified, nonobstructive plaques, as well as spotty calcification plaques.

### The additive effect of MS on plaque type in T2DM

This study highlighted a significant increase in the presence of noncalcified and mixed plaques among MS patients compared to those without MS. It is well documented that mixed or noncalcified plaques correlates strongly with an increased risk of adverse cardiovascular events (25, 26). The elevated occurrence of noncalcified plaques in the MS group may be attributed to heightened inflammatory activity characteristic of MS, which can lead to increased plaque instability. The probable explanation for this greater number of noncalcified/mixed plaques and higher proportion of patients with noncalcified plaques in the MS group may be that MS can increase inflammatory activity and lead to atherosclerotic plaque instability. Furthermore, this study revealed no significant difference in the number of calcified plaques between MS and non-MS groups, which may indicate that in addition to MS, the formation of calcified plaques is also influenced by factors such as age, sex, smoking history, and alcohol consumption history.

Lee et al. revealed that rapid development or progression of CACS, coronary artery stenosis, and vulnerable plaque had a positive correlation with the number of MS components in their long-term follow-up observation (8). Our study had similar results; as the number of MS components increased, so did the proportion of T2DM patients with any noncalcified or mixed plaque and extensive CAD. These findings highlight the need to manage the number of abnormal metabolic markers in patients with diabetes to effectively inhibit the development of coronary atherosclerosis.

### MS increase the presence of high-risk plaque features in T2DM

According to our data, MS was associated with a higher prevalence of spotty calcification in T2DM patients, identifying MS as an independent predictor for this kind of high-risk plaques even after adjusting for confounding factors. It is noteworthy that MS demonstrated a higher OR value in comparison to other established cardiovascular risk factors. Spotty calcification has been more closely linked with acute coronary events than common mixed or non-calcified plaques. According to Ehara S et al, the spotty calcification in plaques is often observed in the culprit lesions of acute myocardial infarction(AMI) patients, suggesting a significant role in clinical outcomes (27). The increased prevalence of these plaques in AMI patients is thought to be due to early he spotty calcification (< 1mm) or microcalcification (< 50mm), which may trigger local tissue stress and lead to plaque instability and subsequent rupture (28, 29).

### MS aggravate extent and severity of CAD in T2DM

Our study demonstrated that T2DM patients with MS are more prone to have extensive and severe CAD. Patients with diabetes typically exhibit a significant burden of coronary plaques. The likey explanation is that diabetes promotes microvascular dysfunction through increased oxidative stress and the production of more proinflammatory substrate, which may faciliatate the formation and progression of plaques (30, 31). This condition is further exacerbated in the presence of MS, which enhances both the occurrence and progression of coronary atherosclerosis (32, 33). MS may facilitate the development of coronary atherosclerosis through multiple mechanisms, one of which is oxidative stress. In patients with MS, insulin resistance and abnormal deposition of adipose tissue elevate oxidative stress and inflammatory responses, which collectively impair endothelial function. And endothelial dysfunction is a key initiating event in the formation of atherosclerotic plaques in the coronary arteries (34, 35).

Furthermore, MS is associated with altered levels of adipose-derived hormones and cytokines, commonly referred to as adipokines. With the progression of obesity, there is an expansion in the volume of cardiac adipose tissue, which tends to accumulate preferentially around the coronary arteries. This phenomenon results in a higher incidence of atherosclerotic plaques in arteries surrounded by perivascular adipose tissue (PVAT). The adipokines secreted by PVAT are known to play a pivotal role in vascular dysfunction, influencing the integrity and function of adjacent blood vessels and promoting the development of atherosclerosis (6). These adipokines not only contribute to vascular inflammation but also to the modulation of arterial remodeling and plaque instability, thereby increasing the risk of cardiovascular events.

### Impact of various components of MS on coronary plaques

Through our multivariate analysis assessing the impact of various components of MS and findings from CCTA, it was evident that hypertension exerted the most significant influence on coronary plaque, followed by obesity. TG and HDL levels showed comparatively less effect. Obesity, historically a core criterion in the diagnosis of MS, plays a crucial role in both the occurrence and development of MS (36). The relationship between obesity, prolonged sedentary behavior, and insulin resistance is well-established. The resultant hyperinsulinemia from these conditions can lead to a cascade of metabolic disturbances, including disrupted glucose metabolism and elevated levels of fatty acids, as well as activation of the sympathetic nervous system, all of which significantly contribute to the onset of cardiovascular diseases (6). Hypertension impacts coronary health by impairing vascular endothelial function, altering wall shear stress, and heightening oxidative stress. These changes initiate a host of pathophysiological responses, including the proliferation of vascular smooth muscle cells, vascular remodeling, and apoptosis, as well as increased cell permeability and expression of adhesion molecules, thereby accelerating the development of atherosclerotic plaques (37, 38). Research by Jiang Yu et al. corroborates the pronounced impact of hypertension on coronary plaque formation in patients with T2DM (39).Given these findings, a stronger emphasis on meticulous blood pressure management in diabetic patients is imperative to mitigate the risk of cardiovascular complications.

### The prevention of coronary artery damage

The current management paradigm for atherosclerotic coronary artery disease (CAD) focuses on comprehensive prevention by addressing major modifiable risk factors (smoking, hypertension, diabetes mellitus, and hypercholesterolemia) while promoting lifestyle modifications through healthy dietary habits, maintenance of normal body weight, and regular physical activity (40). Substantial evidence demonstrates that smoking prevention/cessation, blood pressure normalization, plasma cholesterol reduction, and targeted diabetes management in specific clinical contexts can collectively reduce the incidence of coronary events (41).

## Limitations

Our study has several limitations. First, its cross-sectional design prevents us from interpreting the results as definitive causal relationships, and the mechanism underlying the association between MS and coronary atherosclerotic plaques remains to be clarified. Second, potential confounding factors that may influence lipid levels were not obtained and analyzed in this study, which to some extent limits the scientific rigor of the findings. Third, all participants in this study were recruited from a single hospital center, which may restrict the generalizability of our results.

## Conclusion

MS is independently associated with adverse coronary artery plaque characteristics among patients with T2DM. This association includes a higher prevalence of mixed, noncalcified, nonobstructive, and spotty calcification plaques, along with more extensive overall coronary plaque burden. And among the components of MS, hypertension has the greatest influence on coronary atherosclerosis in DM patients. These findings underscore the critical importance of early detection and effective management of MS in T2DM patients to mitigate cardiovascular risk. Targeted interventions aimed at controlling MS components could potentially reduce the progression of coronary atherosclerosis, thereby improving cardiovascular outcomes in this high-risk population.

## Data Availability

The raw data supporting the conclusions of this article will be made available by the authors, without undue reservation.
